# Dietary L-Tryptophan Modulates the Structural and Functional Composition of the Intestinal Microbiome in Weaned Piglets

**DOI:** 10.3389/fmicb.2018.01736

**Published:** 2018-08-07

**Authors:** Haiwei Liang, Zhaolai Dai, Ning Liu, Yun Ji, Jingqing Chen, Yunchang Zhang, Ying Yang, Ju Li, Zhenlong Wu, Guoyao Wu

**Affiliations:** ^1^State Key Laboratory of Animal Nutrition, China Agricultural University, Beijing, China; ^2^Henan Yinfa Animal Husbandry Co., Xinzheng, China; ^3^Beijing Advanced Innovation Center for Food Nutrition and Human Health, China Agricultural University, Beijing, China; ^4^Department of Animal Science, Texas A&M University, College Station, TX, United States

**Keywords:** L-tryptophan, microbiota, SCFAs, IAA, indole, AhR, intestinal barrier function, weaned piglets

## Abstract

**Background:** Intestinal microbiota plays an important role in regulating metabolism, physiology, and immune response of the host. L-Tryptophan (Trp) are metabolized by several genera of bacteria. It remains largely unknown whether Trp can regulate the composition and diversity of the intestinal microbiota and contribute to intestinal homeostasis.

**Methods:** A total of 126 weaning piglets were fed a corn- and soybean meal-based diet supplemented with 0, 0.2, or 0.4% Trp for 4 weeks. The intestinal microbiota was measured by using bacterial 16S rRNA gene-based high-throughput sequencing methods. Metabolites of Trp and short-chain fatty acids (SCFAs) in the hindgut were determined by high-performance liquid chromatography and gas chromatography, respectively. The mRNA levels for aromatic hydrocarbon receptor (AhR), tumor necrotic factor-α (TNF-α), interleukin-8 (IL-8), and protein abundances of tight junction proteins were determined.

**Results:** Compared with the control group, Trp supplementation enhanced piglet growth performance and markedly altered the intestinal microbial composition as evidenced by enhanced alpha and beta diversity in the microbiome (*P* < 0.05). The abundances of *Prevotella*, *Roseburia*, and *Succinivibrio* genera were enriched, but those of *Clostridium sensu stricto* and *Clostridium XI*, opportunistic pathogens, were decreased with dietary Trp supplementation. Analysis of metabolic pathways indicated enhanced indole alkaloid biosynthesis and Trp metabolism, which was validated by elevated concentrations of 3-indoleacetic acid and indole in the intestinal contents of Trp-supplemented piglets (*P* < 0.05). These changes in Trp metabolites were correlated with activation of AhR and cytochrome p4501 A1 (CYP1A1) in cecum and colonic tissues, and with a decrease in the intestinal mucosal IL-8 mRNA level. Moreover, the protein abundances for zonula occluden (ZO)-1 and occludin were upregulated by Trp supplementation in colonic tissues.

**Conclusion:** Dietary Trp supplementation altered intestinal microbial composition and diversity, improved intestinal mucosal barrier function, activated AhR signaling, and downregulated expression of inflammatory cytokines in the large intestine of weaned piglets. These results indicate a crosstalk between dietary Trp and intestine in nutrition, microbial metabolism, and mucosal immunity.

## Introduction

The intestinal microbiota is now recognized to have broad biological effects on the health and growth of both humans and animals (Blumberg and Powrie, 2012; Lamas et al., 2016). The intestinal microbiota confers several physiological effects to the host by metabolizing dietary nutrients, producing SCFAs from indigestible carbohydrates, synthesis of amino acids and vitamins, regulating metabolism, and affecting the maturation of the intestinal immunity (Turnbaugh et al., 2006; Kamada et al., 2012). These events are fundamental to the integrity of intestinal mucosal barrier function and health of the host (Turnbaugh et al., 2006; Kamada et al., 2012). Importantly, the diversity and composition of the microbial community are influenced by diet, age, stress, lifestyle, and various environmental factors, which in turn would affect, directly or indirectly, nutrient metabolism, immune responses, and intestinal homeostasis (Turnbaugh et al., 2009; Benson et al., 2010; David et al., 2014), thus constituting a cross-talk between intestinal microbiota and host (Zhang et al., 2017). In support of the critical role of the intestinal microbiota, dysbiosis (defined as changes in the intestinal bacterial ecosystem) is associated with intestinal disorders, such as ulcerative colitis, Crohn’s disease, metabolic disease, and malnutrition (Macfarlane et al., 2011; Blumberg and Powrie, 2012). Thus, maintenance and restoration of host intestinal microbiota homeostasis has shown beneficial effects in clinical patients or experimental animals.

Dietary protein intake can alter the gut microbiota, thus contributing to structural and functional changes of the host intestine (David et al., 2014; Beaumont et al., 2017; Kar et al., 2017; Llewellyn et al., 2017; Tilocca et al., 2017). However, there is a scarcity of information regarding impacts of specific amino acids on the gut flora composition and intestinal health in animals. Besides serving as a substrate for protein synthesis, Trp is metabolized to a variety of biologically active compounds, such as serotonin, tryptamine, indoles, kynurenines, and nicotinamide adenine dinucleotide (NAD^+^), which in turn regulate inflammation, immune response, and neurological functions (Yao et al., 2011; Cervenka et al., 2017). Intestinal commensal bacteria can actively metabolize Trp to produce IAA and indole, two critical metabolites that enhance both intestinal mucosal barrier integrity and immune function via activation of the aryl hydrocarbon receptor (AhR) or related signaling pathways (Zelante et al., 2013; Jin et al., 2014; Venkatesh et al., 2014; Lamas et al., 2016; Barratt et al., 2017). These findings highlight a crosstalk between gut bacteria and host intestinal homeostasis in which Trp is a functional amino acid that regulates host physiology and metabolism. Saraf et al. (2017) reported that formula feeding to piglets between day 2 and 21 of age resulted in reduced microbial diversity as well as alterations in Trp metabolism and immune response in the colon. In addition, *in vitro* studies have shown that Trp enhances the abundances of tight junction proteins and modulates protein turnover in intestinal porcine epithelial cells (Wang et al., 2015a). Based on the foregoing, the present study was conducted to test the hypothesis that dietary Trp supplementation can alter intestinal microbial composition and diversity, improve intestinal mucosal barrier function, and activate AhR signaling in the large intestine of weaned piglets.

## Materials and Methods

### Experimental Design and Animals

A total of 126 piglets (Landrace × Yorkshire, initial body weight 7.6 ± 0.04 kg) were randomly assigned into one of three groups (0%, 0.2%, and 0.4% supplemental Trp groups). Each treatment group consisted of six pens with seven piglets per pen. The corn-soybean based diets were formulated to meet nutritional requirements (National Research Council [NRC], 2012) of piglets throughout phase I (7–11 kg BW) (**Supplementary Table S1**) and phase II (11–25 kg BW) (**Supplementary Table S2**), which contained 0.25% and 0.21% Trp, respectively. L-Alanine was used to formulate isonitrogenous diets as previously described (Wang et al., 2008). The amounts of supplemental Trp used in our study were based on a previous study showing that dietary supplementation with 0.23% Trp enhanced feed intake and growth performance in weaned piglets (Jansman et al., 2010). In the present study, all piglets were weaned at 24 days of age and the first day of weaned was recorded as day 0 of the experimental period. At the end of the 28-day trial, 24 piglets (eight from each treatment) were sacrificed by exsanguination. The tissues and the large-intestinal contents were collected and placed in liquid nitrogen and then were stored at -80°C for later analysis.

### DNA Extraction, PCR Amplification, and Bacterial 16S Ribosomal RNA (rRNA) Gene Sequencing

The total genomic DNA of cecal bacteria was extracted by using a DNA Kit (Qiagen, Hilden, Germany) according to the manufacturer’s instructions. The integrity of DNA was assessed by agarose gel electrophoresis, and then the genomic DNA was used as a template for PCR amplification. The 16S RNA V3–V4 gene region was amplified by using the primers F341 and R806 (Sun et al., 2017). PCR amplification was carried out in a 25 μl reaction system and the condition of PCR amplification was: initial pre-denaturation at 94°C for 4 min, denaturation at 98°C for 10 s, renaturation at 58°C for 30 s, elongation at 72°C for 2 min, 30 cycles, and then the last elongation step at 72°C for 10 min. The 16S rRNA gene was sequenced on the Illumina HiSeq PE250 sequencing platform at the Realbio Genomics Institute (Shanghai, China) according to the manufacturer’s instructions.

### Sequence Data Analyses

The sequences raw data obtained from the Illumina HiSeq platform were quality-filtered and demultiplexed by using the Quantitative Insights into Microbial Ecology (QIIME) version 1.8.0-dev (Caporaso et al., 2010), as described by the previous study (Mu et al., 2016). Illumina raw data were treated to remove low quality reads. The reads with an average quality score of no less than 25 and with a length of 400–440 nt were retained by SOAPaligner (v 2.21) (Li et al., 2008). USEARCH version 7.1 software was used for reads cluster and cutoff (based on 97% similar identity) for OTUs, and chimeric sequences were identified and removed using UCHIME (Edgar, 2010). Alpha diversity (chao1, observed species, Shannon index, and Simpson index) was assessed by MOTHUR v.1.35.0 (Schloss et al., 2009). Beta diversity was calculated based on unweighted uniFrac distances by QIIME. An unweighted unifrac PCoA based on OTUs was performed to provide the overview of the microbial diversity and composition in the cecum of pigs fed different doses of tryptophan. Analysis of molecular variance (AMOVA) (Schloss et al., 2009) was performed to compare the difference between different treatment groups. Besides, PCA was used to determine the similarity between the composition of samples under the same condition. LEfSe analysis was performed at the genus level to determine the microbes that had a significant effect on the division of the samples (Segata et al., 2011), and the more intuitive heatmap analysis (Kruskal–Wallis test) was used to indicate similarities and differences in community composition of the samples. PICRUSt was applied to predict the functions of microbiota communities based on the 16S rRNA gene library composition (Langille et al., 2013).

All the sequencing data were submitted to the National Center for Biotechnology Information GenBank Sequence Read Archive database under accession number SRP 150032.

### Measurements of SCFAs by Gas Chromatography

Contents of the cecum and colon were prepared for SCFAs determination as previously described (Peng et al., 2017). Briefly, 1.0 g contents of hindgut were homogenized with 4 ml double distilled water and centrifuged at 12,000 rpm for 10 min at 4°C. Then the supernatant fluid (3 ml) was diluted by 0.6 ml 25% (w/v) metaphosphoric acid solution, the mixture was vortexed and centrifuged at 12,000 rpm for 10 min at 4°C after 30 min incubation on ice. The collected supernatant was filtered through a 0.45 μm polysulfone filter and injected into a HP 6890 Series Gas Chromatograph (Hewlett Packard, PA, CA, United States) for the determination of SCFAs.

### Determination of Trp-Derived Metabolites by High-Performance Liquid Chromatography (HPLC)

Trp-derived metabolites in cecal and colonic contents were extracted with cold 50% methanol (methanol: H_2_O = 1:1) according to the previous study with modifications (Saraf et al., 2017). Briefly, 100 mg samples were weighed and mixed with 700 μl cold 50% methanol. The mixture was homogenized and centrifuged at 12,000 rpm for 10 min at 4°C. The supernatant was transferred to new tubes, mixed with 600 μl cold 50% methanol, and then centrifuged at 12,000 rpm for 10 min at 4°C. The supernatant fluid was mixed with equal volume of working solution (0.12% benzoic acid: 50% methanol = 1:1). The mixture was mixed thoroughly and centrifuged at 12,000 rpm for 10 min at 4°C. The supernatant fluid was filtered through a 0.45 μm polysulfone filter and injected into a Waters e2695 high-performance liquid chromatograph system (Waters, United States) with in-line pre-column derivatization with OPA (Presits and Molnar-Perl, 2003).

### Western Blot Analysis

Colonic tissues were homogenized in liquid nitrogen for protein extraction and the protein abundance was determined with the use of the Western blotting technique as previously described (Wang et al., 2015b). Equal amounts of protein (40 μg) were separated on SDS-PAGE gels, and then proteins were transferred onto PVDF membranes (Millipore, Billerica, MA, United States). The membranes were incubated with 5% skimmed-milk solution in Tris-buffered saline containing 0.05% Tween-20 (TBST) for 30 min at 25°C, and then were incubated with one of the indicated primary antibodies overnight at 4°C. After that, the membranes were washed with TBST three times and then were incubated with horseradish peroxidase (HRP)-conjugated secondary antibody for 1 h. The protein bands were developed by an enhanced chemiluminescence kit (Applygen Technologies Inc., Beijing, China) using the ImageQuant LAS 4000 mini system (GE Healthcare). Quantification of band density was determined by using the Quantity One software (Bio-Rad Laboratories).

### Quantitative Real-Time PCR Analysis

Small pieces of colonic tissues were homogenized in liquid nitrogen. About 30–50 mg of the resulting tissue powders was used for total RNA extraction using the Trizol reagent (Takara, Takara Biomedical Technology in Beijing, China), followed by reverse transcription using the High Capacity cDNA Archive kit (Takara) according to the manufacturer’s protocols. The whole process was carried out under the RNase free condition, and the obtained cDNA and remaining RNA were stored at -80°C for later analysis. SYBR Premix Ex Taq II (TaKaRa) and the ABI-Prism 7500 Sequence Detection System (Applied Biosystems) were used for Real-time PCR. The mRNA level of GAPDH was used as the internal control and the 2^-ΔΔCT^ method was used to determine the fold changes in mRNA levels of different genes in each sample. The primer sequences used for the mRNA determined were listed in **Supplementary Table S3**.

### Statistical Analysis

Experimental data including SCFAs, metabolites, the relative grayscale protein and the relative gene expression, were analyzed by one-way ANOVA and the Duncan multiple comparison test with the use of SPSS statistical software (SPSS, Inc., Chicago, IL, United States). *P* < 0.05 was taken to indicate statistical significance. For the analysis of 16S rRNA gene sequencing data, data were normalized by copy number. In order to compare differences among control and Trp groups, Kruskal–Wallis ANOVA performed on ranks were used for non-parametric data and one-way ANOVA with Bonferroni *post hoc* test were used for parametric data (Mu et al., 2017). *P* < 0.05 was taken to indicate statistical significance.

## Results

### Growth Performance of Piglets

Compared with the control group, dietary supplementation with 0.2% and 0.4% Trp increased (*P* < 0.05) the ADFI of piglets by 9.9% (387 g/d vs. 352 g/d) and 8.5% (382 g/d vs. 352 g/d) from day 0 to day 14. During the whole experimental period, both 0.2% and 0.4% Trp supplementation increased (*P* < 0.05) ADG of piglets by 11.8% (398 g/d vs. 356 g/d) and 6.2% (378 g/d vs. 356 g/d), respectively. However, the FCR was not affected by Trp supplementation (**Supplementary Table S4**).

### Microbiota Composition in the Cecum of Weaned Piglets Supplemented With or Without Trp

**FIGURE 1:**
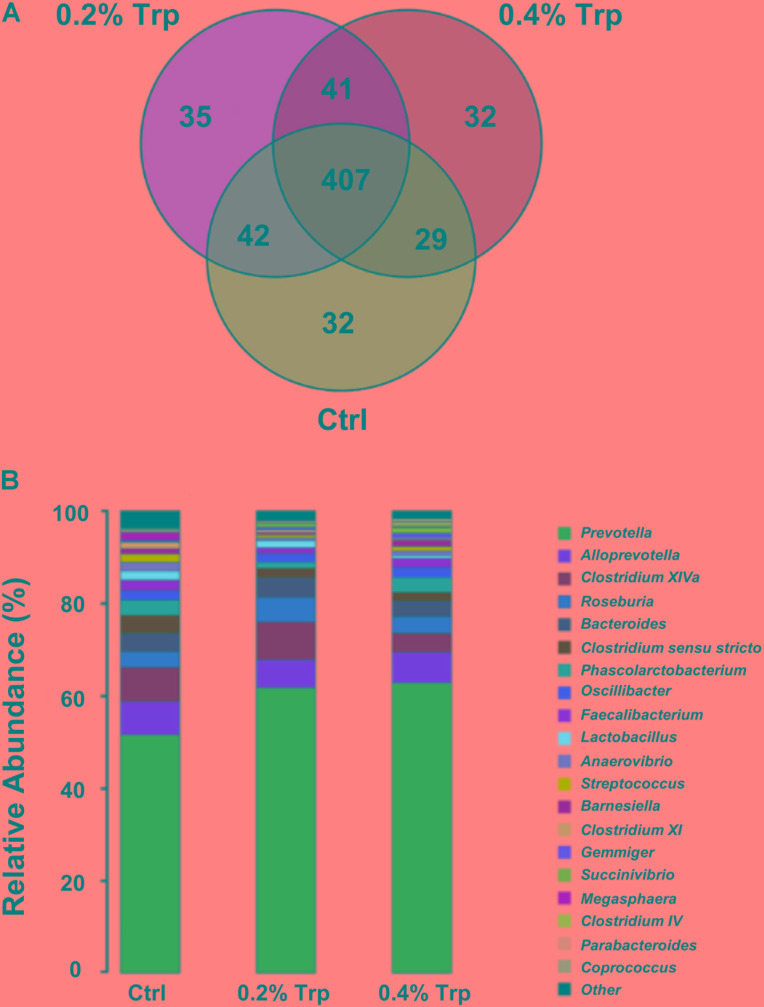
Changes in operational taxonomic units (OTUs) and classification abundance of bacteria. **(A)** The Venn plot of the OTUs in different treatment groups (*n* = 8). **(B)** The relative abundance of the dominant bacteria (percentages) in the cecum contents of weaned piglets at the genus level (*n* = 8). Ctrl, control group; Trp, L-tryptophan.

The microbiota composition in the cecum of weaned piglets was analyzed by the Illumina HiSeq sequencing system. After size filtering, quality control, and chimera checking, a total of 35,050 ± 714, 32,982 ± 1,558, and 34,983 ± 763 reads were observed in the 0%, 0.2%, and 0.4% Trp supplementation groups, respectively. OTUs were obtained at a sequence-similarity level of 97%. A Venn diagram was used to reveal the shared and unique microbiota present in the control and/or Trp supplementation groups. As shown in **Figure 1**, 407 OTUs common to all of the three groups were identified, accounted for 80.4% of all sequences. A varied amount of unique OTUs, 32, 35, and 32 for 0, 0.2, and 0.4% Trp group were observed (**Figure 1A**), respectively. In addition, there are 42, 29, and 41 OTUs shared by 0% and 0.2% Trp groups, 0% and 0.4% Trp groups, and 0.2% and 0.4% Trp groups, respectively.

In agreement with previous studies, *Bacteroidetes* and *Firmicutes* were two of the most abundant phyla at the phylum level in all groups of pigs (**Supplementary Figure S1**). The abundance of *Bacteroidetes* was enhanced by Trp (from 63% in the control group to 71.2% and 69.1% in 0.2% and 0.4% Trp groups, respectively) as compared to the control group. In contrast, the abundance percentage of *Firmicutes* was 35.4%, 27.3%, and 29.3% in 0%, 0.2%, and 0.4% Trp groups, respectively, indicating a reduced abundance of *Firmicutes* as compared with control piglets (**Supplementary Figure S1**).

At the genus level, compared with the control, dietary supplementation with 0.2% tryptophan increased the relative abundance of *Prevotella* (61.7% vs. 51.5%), *Roseburia* (5.31% vs. 3.31%), and *Succinivibrio* (0.64% vs. 0.11%). Of note, dietary supplementation with 0.4% tryptophan reduced the relative abundance of *Clostridium sensu stricto* (1.89% vs. 3.95%), *Clostridium* XI (0.31% vs. 1.33%) and *Lactobacillus* (0.56% vs. 1.95%) (**Figure 1B** and **Supplementary Table S5**). In addition, an 11.8% increase in number of OTUs in the 0.2% Trp group (331 vs. 296 in the control) was observed (**Supplementary Table S5**).

### Changes in the Diversity of the Microbial Community in the Large Intestine

Using the abundances of bacterial OTUs across samples, we explored the global effects of dietary Trp on bacterial diversity in the cecum. As shown, the quantity of observed species increased as the sequencing depth increased. The ends of the rarefaction curves tapered off with increasing numbers of sequencing per sample (**Supplementary Figure S2**), indicating an adequate sequencing depth to investigate the dominant bacterial populations. Chao1, observed species, Shannon index, and Simpson index values were used as parameters of the alpha diversity, which can be used as an indication of the richness of the bacterial community in the intestinal microbiota. Statistical analysis on the alpha diversity at the genus level was conducted (**Supplementary Table S6**). We found that Chao1 index (**Supplementary Figure S2a**) and observed species (**Supplementary Figure S2b**) in the 0.2% Trp group were increased relative to that of the control and 0.4% Trp groups (*P* < 0.05). Although the Shannon index (**Supplementary Figure S2c**) and the Simpson index (**Supplementary Figure S2d**) in the 0.2% Trp group were numerically higher than the control and 0.4% Trp groups, there was no statistically significant difference.

**FIGURE 2:**
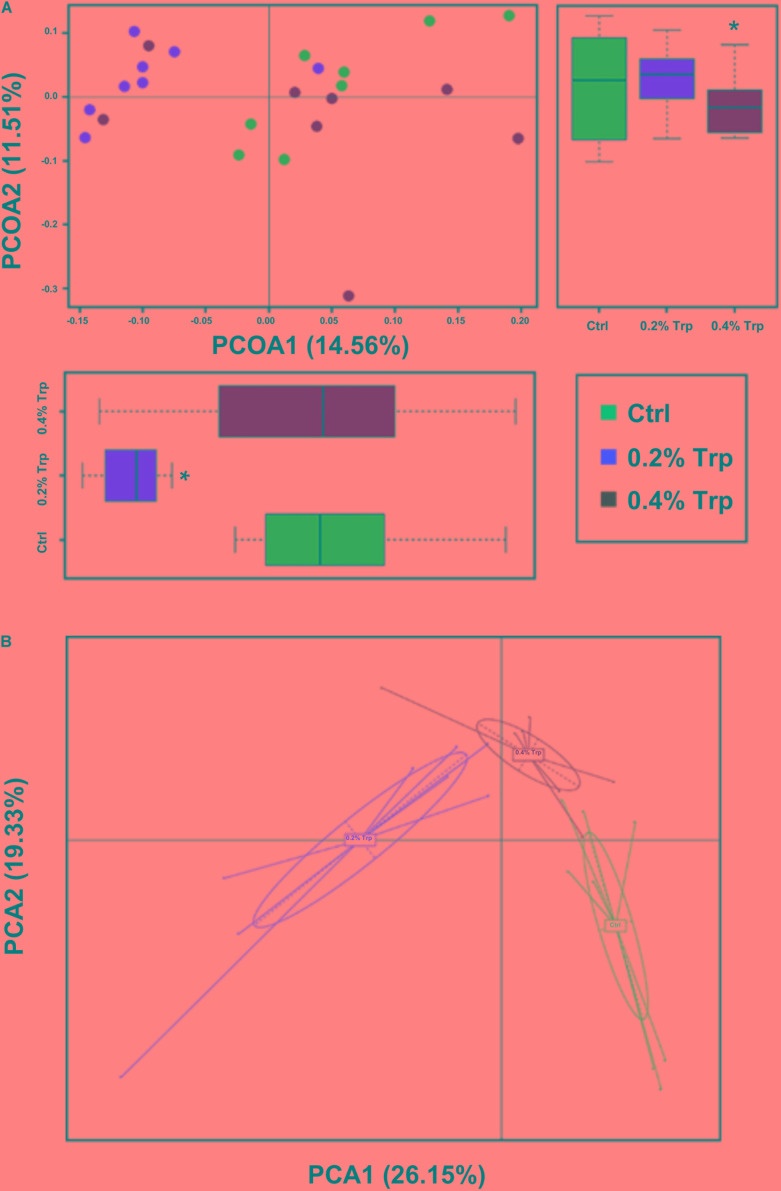
Dietary Trp supplementation altered the beta diversity and overall composition of the microbiota in the cecum. **(A)** The beta diversity of bacteria community in each group, the horizontal and vertical axis scale indicates relative distances (based on the unweighted unifrac distances). PCoA1 and PCoA2 represent the potential factors (Trp supplementation) that caused a microbial composition shift of the cecal samples. Asterisk indicates a statistically significant difference (*P* < 0.05). **(B)** Effects of Trp on the overall composition of the microbiota at the genus level. Principal component analysis (PCA) was based on the OTUs.

Principal coordinate analyses were used to estimate beta diversity among the three treatment groups. The PCoA plot of the unweighted unifrac distances showed that the 0.2% Trp group formed a distinct cluster and separated markedly from the control and 0.4% Trp groups (*P* < 0.05) along the first principal coordinates (**Figure 2A**). Further analysis using the PCA demonstrated that the OTUs of the microbial communities were clustered into 3 groups by Trp supplementation at the genus level (**Figure 2B**).

**FIGURE 3:**
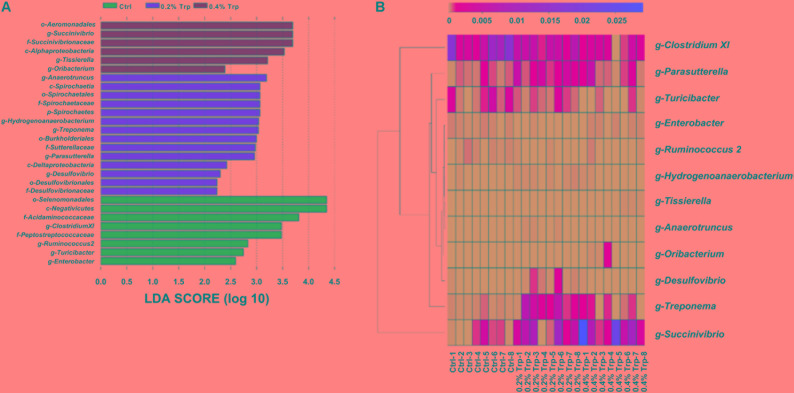
**(A)** Total bacteria in the cecum of the weaned pig which make contributions to the difference at the class, order, family, and genus levels, as analyzed by the LDA effect size (LEfSe) method. **(B)** Heatmap cluster analysis of bacteria at the genus level which have a contribution to group differences (based on a contribution degree at top 12). The heatmap is color-coded based on row z-scores.

According to the results of LEfSe analysis, a total of 28 OTUs at the phylum (1 OTU), class (4 OTUs), order (5 OTUs), family (6 OTUs), and genus levels (12 OTUs) were identified to have significant differences among groups. Among the significantly different OTUs, at the genus level, *Anaerotruncus* and *Treponema* were the two most abundant bacteria in the 0.2% Trp group, as well that for *Succinivibrio* and *Tissierella* in 0.4% Trp group (**Figure 3A**). To compare the relative abundance of the 12 OTUs in all the sequenced samples, the heatmap was used to show the similarities and differences in community composition of the samples (**Figure 3B**). The abundance of *Treponema* in the 0.2% Trp group was 0.0027 which is 25 and 3.7 times higher than that in the control or 0.4% Trp group, respectively. Furthermore, the decreased abundance of *Clostridium* XI and the increased abundance of *Succinivibrio* by dietary Trp supplementation was observed (**Figure 3B**).

### Prediction Analysis of Metabolic Pathways in the Microbiota of the Large Intestine

To investigate the contribution of gut microbiota to host metabolism, the correlation prediction analysis of metabolic pathways was conducted. A total of 11 pathways based on environmental, cellular process, metabolism, and genetic information processing were affected significantly by dietary tryptophan. We found that the significantly higher indole alkaloid biosynthesis, Trp metabolism, and mTOR signaling pathway were affected by Trp (**Supplementary Figure S3**).

### Generation of SCFAs, 3-Indoleacetic Acid, and Indole in the Large Intestine

**FIGURE 4:**
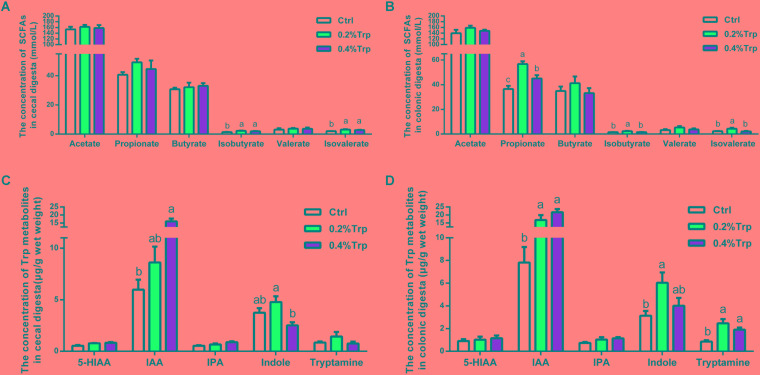
Dietary Trp supplementation promoted the production of SCFAs and tryptophan metabolites in the hindgut of weaned pigs. Data are presented on the concentrations of SCFAs, including acetate, propionate, butyrate, isobutyrate, valerate and isovalerate, in cecal **(A)** and colonic **(B)** contents, as well as the concentrations of Trp-derived metabolites in cecal **(C)** and colonic **(D)** contents. Values are means ± SEMs, *n* = 6. Means without a common letter differ, *P* < 0.05.

To explore whether the change in the intestinal microbiota affects the production of SCFAs following Trp supplementation, the SCFAs of hindgut contents were determined. An elevated concentration of isobutyrate and isovalerate in colonic contents were observed in the 0.2% and 0.4% Trp groups (*P* < 0.05). Also, the amount of propionate in colonic contents was increased in the 0.2% Trp group. However, the amounts of acetate, butyrate, and valerate in colonic contents did not differ among the three groups (**Figures 4A,B**). Moreover, an elevation in the production of IAA was observed in the 0.2% and 0.4% Trp groups relative to the control group. Intriguingly, we found that the production of indole in the 0.2% Trp group was increased (*P* < 0.05) in both the cecum and the colon. The concentrations of tryptamine in colonic contents of the 0.2% and 0.4% Trp groups were higher as compared with that of the control group (*P* < 0.05). In contrast, no difference in the production of 5-HIAA and IPA were observed in the hindgut contents (**Figures 4C,D**). All these data suggested that dietary Trp promoted the generation of SCFAs and Trp metabolites (IAA and indole).

### Activation of AhR and Immune Response

**FIGURE 5:**
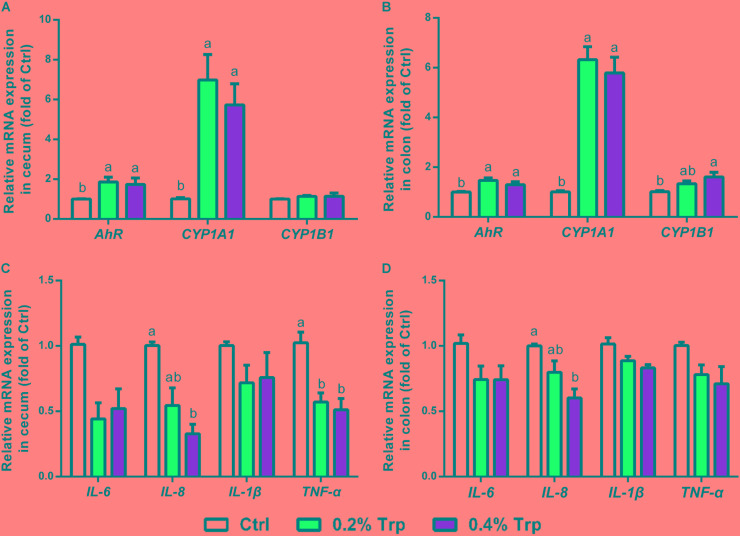
Dietary Trp supplementation influenced *AhR* activation and immune response in the cecum and colon of weaned pigs. The mRNA levels for *AhR*, *CYP1A1* and *CYP1B1* in cecal **(A)** and colonic **(B)** tissues. The mRNA levels for *IL-6*, *IL-8*, *IL-1β* and *TNF-α* are shown for cecal **(C)** and colonic **(D)** tissues. Values are means ± SEMs, *n* = 6. Means without a common letter differ, *P* < 0.05.

Increased production of IAA and indole has been reported as an endogenous ligand responsible for AhR activation (Lee et al., 2017). Therefore, quantitative real-time PCR was performed to determine the mRNA expression of AhR and downstream targets, including CYP1A1, CYP1B1, and various immune cytokines in cecum and colon tissues. As illustrated, the mRNA levels of AhR and CYP1A1 in the cecum and colon epithelium were enhanced (*P* < 0.05) in comparison to the control group. In contrast, CYP1B1 was found to be induced in the colon, but not in the cecum tissues (**Figures 5A,B**). Dietary Trp supplementation reduced the mRNA levels for pro-inflammatory factors (TNF-α and IL-8) in cecum tissues, but reduced IL-8 gene expression in the colon (**Figures 5C,D**). Collectively, Trp supplementation induced AhR activation in both the cecum and the colon, enhanced mRNA levels of CYP1A1 in the hindgut of piglets, and reduced mRNA levels for TNF-α and IL-8 in the cecum.

### Expression of Tight Junction Proteins in the Colon

**FIGURE 6:**
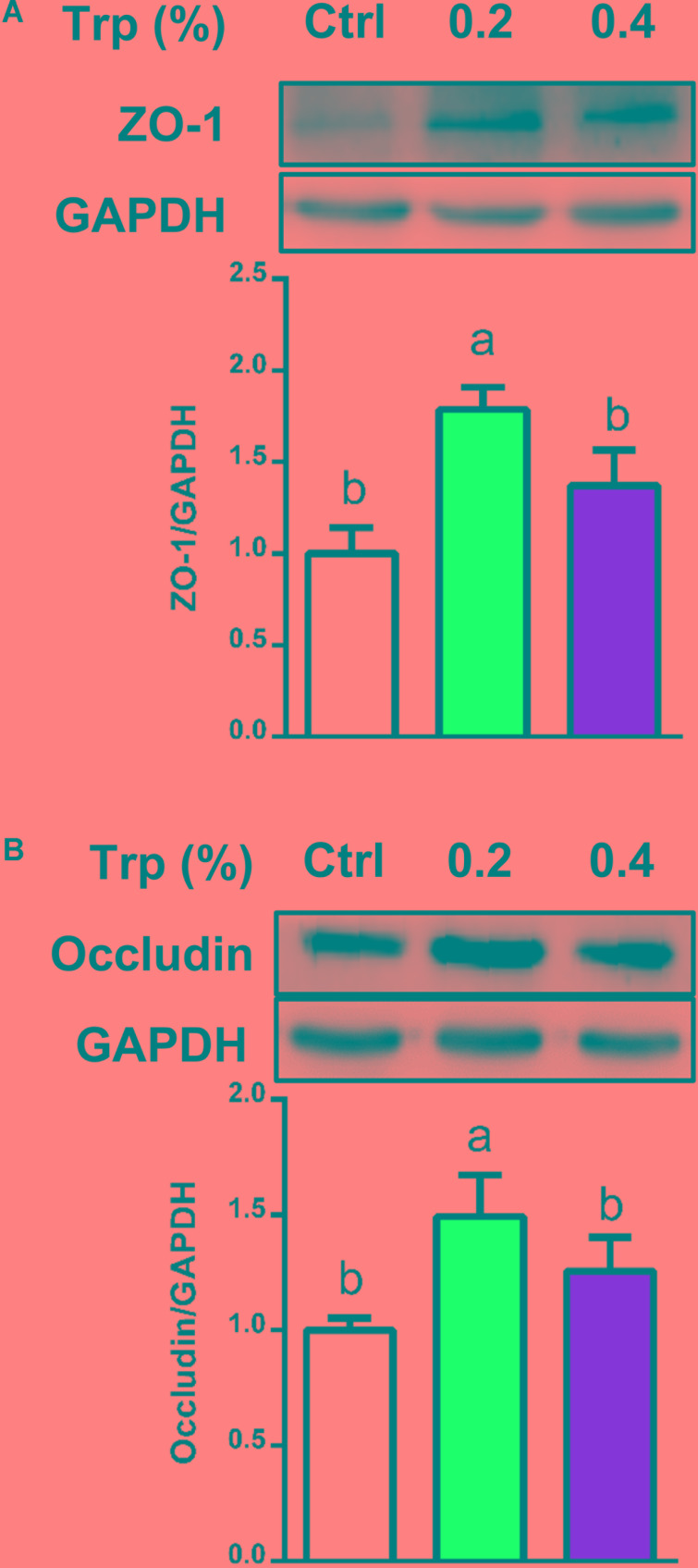
Protein abundances of tight junction in the colon from weaned pigs in 0%, 0.2%, and 0.4% Trp groups. Data are presented on ZO-1 **(A)**, and occludin **(B)**. Values are means ± SEMs, *n* = 6. Means without a common letter differ, *P* < 0.05.

To determine if the alterations in the composition and diversity of microbiota influenced intestinal mucosal barrier integrity, Western blot analysis was performed to investigate the protein abundances for tight junction proteins in colonic tissues of weaned piglets. The results showed that dietary supplementation with 0.2% Trp enhanced the abundances of proteins for ZO-1 and occludin (*P* < 0.05), compared with the control group (**Figure 6**).

## Discussion

A complex and vast microbial community is colonized in the mammalian hindgut which plays an important role in nutritional, physiological, and immunological processes, and profoundly influences intestinal health (Leser et al., 2000; Tsuruta et al., 2016). A large amount of evidence indicates that diet is a critical factor that modulates the gut microbial composition and diversity, regulates immunity response and metabolism, thus exerting a beneficial effect to the host (David et al., 2014; Llewellyn et al., 2017). Of interest, Trp has attracted growing attention due to its functional role in regulating host physiology, metabolism, and immunity (Cervenka et al., 2017; Saraf et al., 2017). However, it remains largely unknown how the intestinal microbiota is modified by Trp supplementation. In the present study, weaning piglets, an animal model for nutrition and metabolism (Guilloteau et al., 2010; Heinritz et al., 2013; Saraf et al., 2017), were supplemented with 0.2% or 0.4% Trp throughout days 35–63 of age. The positive effects of dietary Trp on improving feed intake and growth performance were confirmed in our study. This indicates that the current NRC requirements of growing pigs for Trp have been grossly underestimated. Besides, with the continuous improvement in the technology of mass production of Trp (Ikeda, 2006), the cost of Trp for feed composition will be further reduced in the near future.

To evaluate the effect of Trp on intestinal microbiota, cecal microbiota was extracted for 16S rRNA analysis. In consistence with previous studies (O’Mahony et al., 2015; Yang et al., 2016), we found that *Prevotella* was the dominant bacteria in cecal microbiota among three dietary groups at the genus level, followed by the *Alloprevotella*, *Clostridium XlVa*, and *Roseburia*. Trp supplementation was associated with reduced abundances of *Clostridium sensu stricto*, *Clostridium XI*, *Ruminococcus*, and *Enterobacter*, as well as increased abundances of *Prevotella, Roseburia*, and *Succinivibrio. Clostridium* species are a potential pathogenic bacteria which has been reported to cause intestinal disorders (Wells and Wilkins, 1996). The reduction of these bacteria indicated an inhibitory effect of Trp or its metabolites on potential intestinal pathogens (O’Mahony et al., 2015; Wirthgen et al., 2017; Krishnan et al., 2018). *Prevotella* and *Roseburia* belong to *Bacteroides* and *Firmicutes*, respectively, which have been reported to produce SCFAs, critical molecules with the ability to regulate intestinal homeostasis in humans and animals (Duncan et al., 2006; Pieper et al., 2012; Ellekilde et al., 2014). Accordingly, Trp supplementation increased the concentrations of SCFAs in the large intestine of weaned pigs. Moreover, we found that the concentrations of isobutyrate and isovalerate, BCFAs, produced mainly through microbial deamination of branched-chain amino acids (valine and leucine) (Crown et al., 2015; Neis et al., 2015; Surger et al., 2018), were substantially increased by Trp supplementation, indicating an important role for Trp in stimulating the metabolism of branched-chain amino acids in the hindgut. Further studies are needed to isolate and identify bacteria involved in this process. Noteworthy, although the amount of Trp metabolites produced from the bacterial catabolism of Trp was altered in the large intestine by dietary Trp supplementation, the Trp source for the bacterial fermentation is not clear. And other factors, such as alternation in chemical composition of the ileal digest by Trp, should be also taken into consideration. Therefore, the alternation of the microbiota in the pig large intestine by dietary Trp supplementation can be considered as indirect effect. However, the altered microecology of the large intestine by dietary Trp supplementation will affect intestinal physiology.

Several types of intestinal bacteria, such as *Bacteroides thetaiotaomicron*, *Paracolobactrum coliforme*, *Achromobacter liquefaciens*, *Micrococcus aerogenes*, *Escherichia coli*, *Clostridium difficile*, *Clostridium sticklandii*, *Clostridium lituseburense*, *Clostridium subterminale*, and *Clostridium putrefaciens* have been reported to metabolize Trp into IAA, indole, and other metabolites (Yokoyama and Carlson, 1979; Smith and Macfarlane, 1996; Lee and Lee, 2010). Consistently, these metabolites have been reported to be positively correlated with dietary Trp intake in human subjects and with improved mucosal barrier function (Bansal et al., 2010; Maynard et al., 2012; Hubbard et al., 2015). In our study, the content of IAA in the cecum and colon were elevated by Trp supplementation. Among the bacteria with the ability to produce IAA from Trp, we found that the abundances of *Clostridium XlVa*, *Clostridium IV*, and *Bacteroides* were increased, while the abundances of *Clostridium XI* and *Clostridium sensu stricto* genera were reduced in Trp-supplemented pigs, as compared with controls. The reason for this inconsistence might be due to differences in the capability of bacteria to metabolize Trp and its relative contribution to IAA production among the gut microflora. Also, the existence of other bacteria that can produce IAA from Trp cannot be excluded. For example, bacteria in the genera of *Anaerotruncus*, *Desulfovibrio*, and *Treponema* have been reported to metabolize Trp by tryptophanase and produce indole alkaloid, ammonia, and pyruvate (Yokoyama and Carlson, 1979). In line with the previous study, we observed increases in indole production and the abundance of Trp-producing bacteria in Trp-supplemented pigs. It should be noted that the concentration of indole in the colonic contents of pigs receiving 0.4% Trp supplementation was not altered, which might be due to the limitation of the amount or activity of tryptophanase. Considering that intestinal porcine epithelial cells do not degrade Trp (Chen et al., 2009; Wang et al., 2015a), our results indicate that the production of indole and IAA was predominantly due to Trp-induced alterations in the intestinal microbiota. Besides, we speculate that the production of Trp metabolites by the large-intestinal bacteria may follow a substrate-to-product relationship rather than the changes of the composition and abundance of the Trp-metabolizing bacteria. Moreover, the effects of dietary supplementation of Trp on the composition/abundance of the microbiota in the large intestine either directly or indirectly will be more important as the changed bacteria species together with their metabolites will have important effects on the physiology of the large intestine.

AhR is a cytoplasmic ligand-induced receptor which can be activated by xenobiotics and is associated with their detoxification by activating downstream CYP1A1 targets in various tissues (Linden et al., 2010; Murray et al., 2014). Recent studies demonstrated that certain Trp metabolites can function as endogenous AhR ligands in the gastrointestinal tract, thus regulating immunity response and intestinal homeostasis (Kawajiri et al., 2009; Lee et al., 2011, 2017; Islam et al., 2017). In agreement with these findings, we found that dietary Trp supplementation activated both AhR and the downstream CYP1A1 gene, which might be mediated by bacterial metabolites of Trp. In contrast, the gene expression of CYP1B1 was modestly induced by Trp, suggesting a selective effect of AhR activation on intestinal epithelial cells. This result was consistent with the previous study showing that indole, IAA, tryptamine, and 3-indoxyl sulfate can activate AhR signaling in human CaCo-2 cells (Jin et al., 2014). Furthermore, activation of the AhR signaling participates in resiliency and anti-inflammatory responses (Bansal et al., 2010; Zelante et al., 2013; Fukumoto et al., 2014). This is consistent with our finding that the intestinal mRNA levels for TNF-α and IL-8 were reduced in Trp-supplemented piglets.

Because of the need to provide Trp beyond the current NRC requirements for young pigs, it is important to define a role for this amino acid in intestinal mucosal integrity. Of particular interest, bacterial Trp metabolites have been reported to activate AhR in mucosal tissues and be positively correlated with enhanced protein abundance of ZO-1 and occludin, two critical tight junction proteins involved in intestinal permeability (Peterson and Artis, 2014). This is in agreement with our previous study with intestinal porcine epithelial cells (Wang et al., 2015a) and the novel findings of our present *in vivo* experiments involving weaned pigs. It is noteworthy that the alterations in the composition and diversity of the cecum microbiota by Trp supplementation may be indirect. Besides, Trp itself could enhance the mucosal barrier integrity according to our previous *in vitro* study. Moreover, Trp metabolism by microbiota in the intestine is complex, and results from the present study indicated that Trp metabolites (including indole and 3-indoleacetic acid) or metabolites (SCFAs) from carbohydrates fermentation were associated with intestinal mucosal barrier integrity.

Based on the foregoing, we suggest that Trp administration itself maybe one of the most important factor mediated in the regulation of mucosal barrier integrity. Despite data presented here indicating the role of the microbiota-Trp metabolites-AhR axis, other signaling pathways might be also implicated in the beneficial effects of Trp on intestinal epithelial integrity. For example, farnesoid x receptor (FXR) or pregnane x receptor (PXR) can participate in the regulation of mucosal homeostasis in mice and in the function of cultured hepatocytes (Pavek, 2016; Stepankova et al., 2017). Although we could detect only five major metabolites of Trp in the contents of the piglet large intestine, potential roles of other quantitatively minor products of Trp in gut function cannot be excluded. Besides, in this study, we only analyzed microbiota changes in the large intestine. As dietary Trp was first absorbed and metabolized in the small intestine, therefore, further study to uncover the alteration of microbiota community in the small intestine by dietary Trp is needed.

## Conclusion

Using the piglet as an animal model, we demonstrated that dietary Trp supplementation for 4 weeks altered the intestinal microbiota and enhanced the abundance of intestinal tight-junction proteins. The beneficial effect of Trp was associated with an increased abundance of microflora and consequent activation of AhR signaling. Modification of the intestinal microbiota by Trp or its metabolites might be a novel strategy to improve intestinal health in humans and animals.

## Ethics Statement

All animal treatment and experimental procedures were approved by the China Agricultural University Animal Care Committee.

## Author Contributions

GW and ZW designed the research. HL, ZD, and JL conducted the research. HL, NL, YJ, JC, YZ, JL, and GW analyzed the data. HL, ZD, YY, ZW, and GW wrote the paper. ZD, ZW, and GW had responsibility for the final content. All authors read and approved the final manuscript.

## Conflict of Interest Statement

JL was employed by Henan Yinfa Animal Husbandry Co. The remaining authors declare that the research was conducted in the absence of any commercial or financial relationships that could be construed as a potential conflict of interest.

## Supplementary Material

FIGURE S1 The relative abundances of microbiota at phylum **(a)**, class **(b)**, order **(c)**, and family **(d)** levels.

FIGURE S2 Alpha diversity of each sample in the cecum of weaned pigs. Alpha diversity of bacterial community in each sample was reflected by the Chao1 index **(a)**, Observed species **(b)**, the Shannon index **(c)**, and the Simpson index **(d)**.

FIGURE S3 The correlation prediction analysis of metabolic pathways in the large intestine of Trp-supplemented pigs.

TABLE S1 Ingredients and chemical composition of experimental diets (day 0–14). The basal diets of phase I (7–11 kg BW of piglets). BW, body weight; DE, digestible energy; CP, crude protein.

TABLE S2 Ingredients and chemical composition of experimental diets (day 15–28). The basal diets of phase II (11–25 kg BW of piglets).

TABLE S3 Primer sequences used for PCR analysis.

TABLE S4 Effects of dietary Trp supplementation on the growth performance of weanling piglets. Piglets were weaned at 24 days of age and the first day of weaned was recorded as day 0. ADG, average daily gain; ADFI, average daily feed intake; FCR, feed conversion ratio. Means without a common letter differ, *P* < 0.05.

TABLE S5 Effects of dietary Trp on the cecal microbiota at the genus level in weaned piglets. Values are means and SEMs, *n* = 8. Means without a common letter differ, *P* < 0.05.

TABLE S6 Effects of dietary Trp on the alpha diversity of the cecal microbiota in weaned piglets. Values are means and SEMs, *n* = 8. Means without a common letter differ, *P* < 0.05.

## Abbreviations

5-HIAA: 5-hydroxyindole-3-acetic acid
ADFI: average daily feed intake
ADG: average daily gain
AhR: aromatic hydrocarbon receptor
BCFAs: branched-chain fatty acids
CYP1A1: cytochrome p4501 A1
CYP1B1: cytochrome p4501 B1
FCR: feed conversion ratio
HPLC: high-performance liquid chromatography
IAA: indole-3-acetic acid
IL-8: interleukin-8
IPA: indole propionic acid
LEfSe: LDA effect size
mTOR: mammalian target of rapamycin
NRC: National Research Council
OTUs: operational taxonomic units
PCA: principal component analysis
PCoA: principal coordinate analyses
SCFAs: short-chain fatty acids
TNF-α: tumor necrotic factor-α
Trp: L-tryptophan
ZO: zonula occluden
