# Supplementary material for: Dietary L-Tryptophan Modulates the Structural and Functional Composition of the Intestinal Microbiome in Weaned Piglets
Source: Front Microbiol. 2018 Aug 7;9:1736. doi: 10.3389/fmicb.2018.01736 (PMC6090026; doi:10.3389/fmicb.2018.01736)
Supplement: TABLE S1 — Ingredients and chemical composition of experimental diets (day 0–14). The basal diets of phase I (7–11 kg BW of piglets). BW, body weight; DE, digestible energy; CP, crude protein. [file Table_1.PDF]

SUPPLEMENTAL TABLES

Table S1: Ingredients and chemical composition of experimental diets (day 0-14)

Items	Diet		
	Ctrl	0.2% Trp	0.4% Trp
Ingredients%			
Corn	46.2	46.2	46.2
Extruded-corn	12	12	12
Soybean meal (46%)	15.31	15.31	15.31
Extruded-soybean	10	10	10
Fish meal (63.0%)	4	4	4
Whey powder	6.5	6.5	6.5
Cornstarch	0.064	0.039	0.013
Soybean oil	2	2	2
L-Lysine (99.0%)	0.15	0.15	0.15
Trp (98.0%)	0	0.204	0.408
L-Alanine (99.8%)	0.446	0.267	0.089
Limestone powder	0.81	0.81	0.81
Dicalcium phosphate	1.12	1.12	1.12
Salt	0.4	0.4	0.4
1% Premix	1	1	1
Total	100	100	100
Nutrient levels % (Theoretical value)			
DE (Mcal/kg)	3.52	3.52	3.52
CP	19.49	19.49	19.49
L-Lysine	1.18	1.18	1.18
L-Methionine + L-Cysteine	0.68	0.68	0.68
L-Threonine	0.74	0.74	0.74
Trp	0.25	0.44	0.64
L-Isoleucine	0.75	0.75	0.75
Nutrient levels % (Chemical analysis)			
Total energy (Mcal/kg)	4.05	4.02	4.02
CP	20.62	20.12	20.15
Crude ash	5.3	5.8	5.3

Table S2: Ingredients and chemical composition of experimental diets (day 15-28).

Items	Diet		
	Ctrl	0.2% Trp	0.4% Trp
Ingredients%			
Corn	55.25	55.25	55.25
Extruded-corn	12	12	12
Soybean meal (46%)	13.51	13.51	13.51
Extruded-soybean	10	10	10
Fish meal (63.0%)	3.28	3.28	3.28
Cornstarch	0.064	0.039	0.013
Soybean oil	2	2	2
L-Lysine (99.0%)	0.15	0.15	0.15
Trp (98.0%)	0	0.204	0.408
L-Alanine (99.8%)	0.446	0.267	0.089
Limestone powder	0.74	0.74	0.74
Dicalcium phosphate	1.17	1.17	1.17
Salt	0.4	0.4	0.4
1% Premix	1	1	1
Total	100	100	100
Nutrient levels % (Theoretical value)			
DE (Mcal/kg)	3.51	3.51	3.51
CP	18.41	18.41	18.41
L-Lysine	1.07	1.07	1.07
L-Methionine + L-Cysteine	0.65	0.65	0.65
L-Threonine	0.68	0.68	0.68
Trp	0.21	0.41	0.60
L-Isoleucine	0.69	0.69	0.69
Nutrient levels % (Chemical analysis)			
Total energy (Mcal/kg)	4.05	4.04	4.05
CP	18.64	18.63	18.62
Crude ash	4.5	4.8	4.7

Table S3: Primers sequences used for PCR.

Gene	Primer sequence (5'- 3')
AhR-F	TGAAAACCAAAAGCCTGGAC
AhR-R	TCCTCCGCTCTGAAACTTGT
CYP1A1-F	CCTTCACCATCCCTCACAGT
CYP1A1-R	ATCACCTTTTCACCCAGTGC
CYP1B1-F	AATAACGGGGGAAATTCCTG
CYP1B1-R	CACCGAAACACAATGCAATC
IL-6-F	TGTCGAGGCTGTGCAGATTA
IL-6-R	GCATTTGTGGTGGGGTTAGG
IL-8-F	GTGATTGAGAGTGGACCCCA
IL-8-R	CCTTCTGCACCCACTTTTCC
IL-1β-F	TGAATTCGAGTCTGCCCTGT
IL-1β-R	AGTCCCCTTCTGTCAGCTTC
TNF-α-F	GGCCCAAGGACTCAGATCAT
TNF-α-R	GCATACCCACTCTGCCATTG
GAPDH-F	CTCGGAGTGAACGGATTTGG
GAPDH-R	AGTGGAGGTCAATGAAGGGG
341-F	CCTACGGGRSGCAGCAG
806-R	GGACTACVVGGGTATCTAATC

Table S4: Effects of dietary tryptophan supplementation in the growth performance of weanling piglets.

Items	Diet			SEM	*P* value
	Ctrl	0.2% Trp	0.4% Trp		
Initial BW, kg	7.67	7.61	7.69	0.04	0.698
Day 14 BW, kg	10.9[b]	11.3[a]	11.3[a]	0.07	0.004
Day 28 BW, kg	17.6[b]	18.8[a]	18.3[a]	0.16	0.004
ADG, g					
Day 0 to 14	228[b]	264[a]	260[a]	5.02	0.001
Day 15 to 28	484[b]	533[a]	496[ab]	8.76	0.044
Day 0 to 28	356[b]	398[a]	378[a]	5.91	0.005
ADFI, g					
Day 0 to 14	352[b]	387[a]	382[a]	5.94	0.026
Day 15 to 28	888	894	889	13.57	0.983
Day 0 to 28	620	640	635	8.21	0.598
FCR					
Day 0 to 14	1.55	1.47	1.47	0.02	0.136
Day 15 to 28	1.84	1.69	1.79	0.03	0.209
Day 0 to 28	1.74	1.61	1.68	0.26	0.126

Table S5: Effects of dietary tryptophan on cecal microbiota at genus level in weaned piglets.

Genus level of bacteria	Diet			SEM	*P* value
	Ctrl	0.2% Trp	0.4% Trp		
Prevotella	51.50[b]	61.70[a]	62.70[a]	1.60	0.002
Alloprevotella	7.25	6.08	6.62	0.42	0.540
Clostridium XlVa	7.40	8.17	4.15	0.93	0.180
Roseburia	3.31[b]	5.31[a]	3.52[b]	0.27	0.001
Bacteroides	3.97	4.20	3.39	0.67	0.888
Clostridium sensu stricto	3.95[a]	2.11[b]	1.89[b]	0.31	0.007
Phascolarctobacterium	3.29[a]	1.27[b]	3.20[a]	0.37	0.031
Oscillibacter	2.04	1.83	2.21	0.24	0.824
Faecalibacterium	2.30	1.38	1.98	0.54	0.793
Lactobacillus	1.95[a]	1.51[a]	0.56[b]	0.26	0.082
Anaerovibrio	1.83	0.64	1.02	0.63	0.748
Streptococcus	1.84	0.61	0.96	0.51	0.620
Barnesiella	1.18	0.46	1.41	0.24	0.237
Clostridium XI	1.33[a]	0.61[b]	0.31[b]	0.15	0.012
Gemmiger	0.39	0.56	1.14	0.21	0.347
Succinivibrio	0.11[b]	0.64[ab]	1.32[a]	0.18	0.015
Megasphaera	1.59	0.04	0.21	0.51	0.410
Clostridium IV	0.20	0.27	0.77	0.20	0.474
Parabacteroides	0.37	0.40	0.33	0.08	0.953
Coprococcus	0.29	0.13	0.46	0.07	0.164
Other	3.90	2.10	1.81	0.44	0.102
OTUs	296[b]	331[a]	289[b]	7.47	0.038

Table S6: Effects of dietary tryptophan on alpha diversity of cecal microbiota in weaned piglets

Items	Diet			SEM	*P* value
	Ctrl	0.2% Trp	0.4% Trp		
Chao1	315[b]	372[a]	320[b]	9.50	0.016
Observed species	265[b]	305[a]	257[b]	7.54	0.015
Shannon	5.58	5.90	5.38	0.10	0.082
Simpson	0.95	0.96	0.94	0.004	0.110
